# Perceived Time, Frequency, and Intensity of Engagement and Older Masters Athletes' Subjective Experiences

**DOI:** 10.3389/fspor.2021.653590

**Published:** 2021-05-25

**Authors:** Sarah Deck, Alison Doherty, Craig Hall, Angela Schneider, Swarali Patil, Glen Belfry

**Affiliations:** ^1^School of Kinesiology, Faculty of Health Sciences, Western University, London, ON, Canada; ^2^Department of Medicine, University of Alberta, Edmonton, AB, Canada

**Keywords:** older adults, sports, benefits, training, competition

## Abstract

Masters athletes are a unique group of older adults whose experiences may provide valuable insights into the role of sport for successful aging. The purpose of this study was to explore whether masters athletes' social and psychological experiences vary with their time, frequency, and perceived exertion in training and competition. Semi-structured interviews were conducted with 40 men and women older masters athletes, aged 50–79 years (M = 66), who were active at the competitive level across a variety of sports (e.g., volleyball, curling, rowing, dragon boating, running, swimming, and basketball) at the time of the study. Results indicate that all participants experienced social and psychological benefits from engaging in masters sport. Only the high-frequency engagement subgroup (participating five to seven times per week in training and/or competition) reported social downsides, in terms of missing time with family and friends outside of masters sport. However, some participants described the positive family support (e.g., spouse who endorses sport participation) that overrides some of the social costs. These findings have implications for realizing positive experiences with minimal engagement in masters sport, yet an apparent threshold of participation beyond which negative social consequences may be experienced. This is an important consideration for the design and promotion of sport for older adults.

## Introduction

The rate of sport participation continues to steadily decline with age in several countries, including Canada (Statistics Canada, 2020). Despite this steady drop in participation, there is a unique cohort—and upward trend (Dionigi et al., 2018)—of adults who continue with competitive sport past the typical age for top performance (Weir et al., 2010; Dionigi et al., 2013). Individuals who continue to train and participate in athletic competitions designed for older participants are masters athletes. Eligibility for masters competition is based on age, with the minimum qualifying category as young as 30 years, although this differs across sports (e.g., swimming, athletics, and basketball; Weir et al., 2010). The focus of this paper is masters athletes aged 50 years and older; a cohort that Dionigi (2006) and Dionigi et al. (2018) distinguish as “older” (vs. younger and mid-life) masters athletes, and an age that represents a critical point for reduced cardiac output (Nikolaidis et al., 2018).

Masters athletes typically maintain higher than average levels of physical activity in comparison to their same-age peers through training and competition (Baker et al., 2010). Geard et al. (2017) present a strong case for *older* masters athletes being exemplars of successful aging, as they may derive a variety of benefits directly from their sport engagement (Kusy and Zieliński, 2006). There is value, then, in better understanding who these masters athletes are, what they do, and their sport experiences. Geard et al. define successful aging as a late-life process of change characterized by high functioning across the physical, psychological, cognitive, and social domains. They argue that research is needed to better understand the successful aging benefits derived from competitive sport.

The physical benefits of training for older adults, including greater strength and power (Seguin and Nelson, 2003), and improved cardiorespiratory fitness (Nakamura et al., 2007), are well-known. Moreover, there is recent evidence that masters athletes also have an attenuated aging profile associated with preserved DNA sequencing when compared to age-matched non-athletes (Aguiar et al., 2020). Regular strength training (defined as three times per week) by older adults in general has been shown to not only produce expected benefits such as building muscle strength and mass, and preserving bone density, but also prolong independence and increase vitality (Seguin and Nelson, 2003). In a review, Bouaziz and colleagues (2016) found that combining different modalities of training (multi-component training; e.g., endurance and strength training) improved not only fitness and metabolic outcomes such as diabetes, along with improved cognitive performance, but also overall feelings of well-being (Bouaziz et al., 2016). Evidence suggests that older adults' participation in competitive sport may result in better physical health (i.e., better sleep patterns, better diet, and healthier lifestyle overall; Shephard et al., 1995). Recently, Geard et al. (2018, 2020) established that masters athletes have higher physical functioning (fewer physical limitations in daily life) than adults of similar ages who do not participate in competitive sport. While masters athletes have higher rates of injury with their competitive sport participation, they are likely to have much lower rates of chronic disease compared to their less active counterparts (Patelia et al., 2018).

Psychological and social benefits of older adults' sport participation have also been highlighted (Eime et al., 2013), including greater meaning to life, enjoying the competitive challenge, and meeting new friends (Smith and Storandt, 1997; Ogles and Masters, 2000; Dionigi et al., 2011; Young et al., 2018). There is also some evidence suggesting that participation by older adults in competitive sport may result in more prosocial behavior, passion, community involvement, and travel benefits (Lyons and Dionigi, 2007; Dionigi et al., 2011). These social benefits also extend into athletes' homes, as family members have been reported to play an important role in supporting the continued participation of these athletes (Hodge et al., 2008; Young and Medic, 2011; Dionigi et al., 2012). Athletes have reported training with their spouse and children as beneficial to bringing their families closer and a reciprocal effect on each other's participation in sport (Dionigi et al., 2012). As with the physical component, potential negative psychological and social consequences of masters sport engagement have been identified, including family opposition, negative emotions, difficulty maintaining social relationships, burnout, and regret (Dionigi et al., 2012; Young, 2013; Young et al., 2015; Appleby and Dieffenbach, 2016).

Research further indicates that physical benefits can be positively affected by the frequency, intensity, and volume of training by older adults (Maharam et al., 1999). However, to our knowledge, there has been no consideration of any possible effect of training time or frequency, or perceived intensity on subjective psychological and social experiences. Understanding the nature of older adults' sport training and competition that may promote experiencing positive benefits or avoiding the downsides of participation can be an important contribution to insights into successful aging.

The present study aimed to build on the existing body of research and to address the call for qualitative studies on masters athletes that consider aspects that have previously been considered only quantitatively (i.e., frequency, intensity and time; Dionigi, 2006). Therefore, the purpose of the present study was to explore whether time, frequency, and perceived intensity of training and competition align with psychological and social experiences of older masters athletes (50+). We used a cross-sectional qualitative research design to achieve this purpose.

## Methods

### Participants and Context

Semi-structured interviews were conducted with masters athletes who, following IRB approval, were recruited based on the following inclusion criteria: (a) must be 50 years of age and older, (b) must be able to read and write in English in order to give consent, and (c) must be actively competing at any level in masters sport (regional, national, and international). These criteria ensured our focus on the cohort of older masters athletes (Dionigi, 2006; using the usual 50–60 year age category as the lower limit; Weir et al., 2010) who were competitive athletes. Interviewees were recruited through local masters teams and clubs in Ontario, Canada, and through Masters Ontario (the provincial sport governing body for masters sport). Coaches and club managers were first contacted to distribute recruitment posters. For interested athletes, recruitment information included contact information for the researchers in order to schedule an interview. After connecting with prospective participants, interview times were set with individuals interested in participating in the study.

A total of 40 masters athletes (19 women, 21 men) were interviewed individually by one of two research assistants, with each session ranging from 22 to 75 min (M = 48 min). The athletes were engaged with 15 different sports (with some athletes competing in more than one): volleyball (*n* = 4), dragon boat racing (*n* = 3), tennis (*n* = 1), running (*n* = 6), triathlon (*n* = 2), swimming (*n* = 14), rowing (*n* = 2), track and field (*n* = 2), curling (*n* = 2), basketball (*n* = 3), ice hockey (*n* = 1), squash (*n* = 1), cycling (*n* = 2), and slo-pitch (*n* = 1). At 40 interviews, we determined that saturation was reached (Hennink et al., 2017; Braun and Clarke, 2019), as we were hearing consistent descriptions of a range of training and competition engagements, and a variety of social and psychological experiences. Participants were interviewed over the phone or in person at a location that was most convenient for them (e.g., the University lab or local sports club/practice area). The interviews were transcribed verbatim and participants were then invited by email to review the transcription at their convenience and provide any corrections to ensure that the information was conveyed in its intended manner (Doyle, 2007). The profile of athletes by age, gender, hours, and frequency of training and competition, and perceived intensity is presented in Table 1.

**Table 1:** Participant profile (age, gender, training/competition frequency [times/week], training/competition time [hours/week], and perceived intensity [/10]).

**Variable**	**Frequency**	**%**	**Mean (SD)**	**Median**	**Range**
Age (years)			66.34 (7.7)	67	50–79 years
Gender					
Women	19	47.5%			
Men	21	52.5%			
Frequency^*^ (times/week)			4.6 (1.6)	4	1–7
1–2/week	5	12.5%			
3–4/week	15	37.5%			
5–7/week	19	47.5%			
Hours (per week)			5.2 (3.2)	3	1–12
Perceived intensity					
Training (/10)			5.3 (1.2)	5	1–10
Competition (/10)			8.5 (1)	10	3–10

* *Frequency includes number of training and/or competition sessions per week*.

### Interview Guide

The semi-structured interview guide was developed by the authors. It enabled the interviewers to guide the discussion while exploring emergent topics and allowed the participants more freedom to answer openly (Rubin and Rubin, 1995). In addition to providing background information about their age, gender, and sport involvement, participants were asked about their time (hours) and frequency (number of sessions) of training and competition per week. They were also asked to describe their perceived level of exertion in training and in competition using a scale from 1 (*no exertion*) to 10 (*maximal exertion*). Participants were then asked about the social and psychological experiences associated with their engagement in masters sport. Specifically, they were asked to describe the following: “How do you feel being a competitive masters athlete has benefitted you socially?,” “How has it benefitted you psychologically?,” “What are the social downsides of being a competitive masters athlete?,” and “What are the psychological downsides?” Probes were used to further explore athletes' responses, as the semi-structured design allowed for flexibility and reflexivity to explore some topics that varied between participants in the very conversational approach (Patton, 2015).

### Data Analysis

The entire research team composed of physically active men and women—including three who are older masters athletes—was involved in data analysis. The first step was organizing the data (Gibson and O'Connor, 2003). Each interview was transcribed into Microsoft Word and imported into NVivo8^©^. Transcripts from those participants who took the opportunity to review and change their document to reflect their true thoughts were downloaded and used to replace the original transcript. Our analytical framework was based on previous research on the social and psychological experiences of masters sport participation—specifically, psychological benefits, social benefits, psychological downsides, and social downsides (Dionigi, 2017)—which were presented in the interview questions. Thus, responses to “How do you feel being a competitive masters athlete has benefitted you (socially, psychologically)?” were coded as “Social Benefits” and “Psychological Benefits,” respectively. We applied the process of convergence and divergence in coding to ensure data belonged in one theme and not another (Patton, 2015). This was also helpful to highlight the core meanings of each theme (Patton, 2015). The first author read and coded all 40 transcripts, with the remaining authors reading and coding at least four transcripts each. Very few discrepancies in coding and description of core meanings were indicated, and these were discussed and reconciled by the team (inter-rater reliability; Smith and McGannon, 2018), enriching the overall analysis and findings (Gibson and O'Connor, 2003).

In order to determine whether the participant characteristics of interest aligned with the perceived social and psychological benefits and downsides of masters sport, the athletes were first grouped by frequency of engagement into subgroups of one to two sessions per week (*n* = 5), three to four sessions per week (*n* = 15), and five to seven sessions per week (*n* = 19) for further analysis. To better understand the athletes' pattern of engagement (i.e., individuals may train only once a week for 5 h or five times a week for 1 h), a correlation analysis was conducted. It revealed that frequency of sessions and time or hours per week committed to training and competition were highly correlated (*r* = 0.73, *p* < 0.001). Thus, time was not employed as a separate grouping variable and the focus was on number of sessions per week. Perceived exertion in training ranged from as low as 1 (no exertion) to as high as 10 (maximal exertion) on the 10-point scale. There was very little variation among participants, with 70% indicating an intensity level of 4–6/10 for training (overall M = 5.3, median = 5). There was even less variation among participants in perceived exertion in competition, with 70% indicating an intensity level of 8–10 when competing (overall M = 8.5, median = 10). Thus, perceived intensity of engagement was not employed as a grouping variable for further analysis. Subjective experiences were not distinguished for training vs. competition during the interviews and so further analysis based on different intensity in these masters sport contexts was not possible.

## Results

Variation in the participants' psychological and social experiences with masters' sport by frequency of engagement are described below (and in Table 2), along with representative quotations of numbered participants. There was evidence of psychological and social benefits, and social downsides only, with no indication of psychological downsides among the sample of participants.

**Table 2:** Frequency table of older masters athletes' who perceived benefits and downsides to participation, by frequency of training/competition sessions.

	**1–2 times per week (*n* = 5)**	**3–4 times per week(*n* = 15)**	**5–7 times per week (*n* = 19)**
Psychological benefits (stress relief, greater self-confidence, sense of pride)	5 (100%)	15 (100%)	19 (100%)
Social benefits (positive relationships with family and with masters sport friends)	5 (100%)	15 (100%)	19 (100%)
Social downsides (missed family time and events, change in circle of non-sport friends)	0	0	16 (84%)
Psychological downsides	0	0	0

### Psychological Benefits

All masters athletes, whether they were engaged in their sport one to two times per week (*n* = 5), three to four times per week (*n* = 15), or five to seven times weekly (*n* = 19), experienced positive psychological benefits, described as stress relief, greater self-confidence, and a sense of pride. Participant 26 (one to two times per week) shared that, “I get away from the nonsense around the house, so mentally I think I get a break from the everyday run of the mill type tasks and crazy things that are going on.” Similarly, Participant 6 (three to four times per week) indicated that, “I think it's important for mental health and staying resilient.” Participant 17, who was engaged five or more times per week, said, “It's a distraction and a stress release. It's a huge coping mechanism for me, which isn't maybe healthy but it's what I do.” Their masters sport involvement was also reported to “help our self esteem [since] we're still able to compete and play well at our age and it helps the self-image and things like that” [Participant 25 (five to seven times per week)]. Other participants shared that it is “fun to test yourself to see if you do or don't make shots, and if it does happen, I overcame … and I am proud of myself for having done it” [Participant 3 (three to four times per week)], and “Just to win at something, to succeed, that's satisfying” [Participant 14 (one to two times per week)].

### Social Benefits

Similarly, all masters athletes in each of the subgroups indicated experiencing social benefits as a result—and regardless of the frequency—of their engagement. The social benefits were described as positive relationships with both family and friends in masters sport. As Participant 1 (three to four times per week) shared, with a laugh, “My kids think I rock now!” For Participant 26 (one to two times per week), the positive relationships were founded on enjoying sport with family: “Ya, my kids are involved as well a little bit with volleyball, so we have fun when we do get together, we play.” A masters triathlete shared that, “My son does triathlons. My grandkids do triathlons. Now I have four great grandkids; 2 of them … they've done triathlons, so they're all into [the] sport” [Participant 5 (five to seven times per week)]. Additionally, Participant 19 (one to two times per week) noted that, “Socialization is a big part, we are all friends, it's kind of like family. There is nothing we wouldn't do to help each other out.” Participant 39 (three to four times per week) shared that, with not participating in masters sport “I probably would miss the people that I would see and the type of people they are. I think we, as a group, have a more positive outlook.” Participant 34 (three to four times per week) said:

It's possible that swimming isn't the best social sport, but a number of us often go out for breakfast after. I usually try to go on Mondays and Wednesdays, so 6 or 7 of us go out to the local restaurant here and have breakfast and chat and there is usually a good discussion, these are people from different backgrounds with different views and so on.

### Social Downsides

The masters athletes who trained and competed five or more times per week were the only ones to indicate some negative impact from their sport engagement, with 16 of the 19 participants identifying this experience. They described missing family time and functions due to their sport schedule and a change in their circle of friends due to time spent with those involved in their sport. Participant 32 (five to seven times per week) discussed that, “It's changed my friendships I guess, because I dedicate so much time to this activity … my lifelong friends, they don't really get it.” The negative consequences to relationships of greater engagement in one's sport was described by Participant 4 (five to seven times per week) who stated, “…it takes time and you may find that you are putting this ahead of other things.” Participant 21 (five to seven times per week) reiterated this by discussing how family sometimes does not understand the commitment:

My family from [another city] was here … and I was going to the pool and they were like “why do you have to go? '…' why do you have to go for three hours?” So, they don't fully understand my commitment and how important it is to me. So, there are times where that happens.

Participant 37 (five to seven times per week) agreed: “You know doing an Iron Man is tough, tough on family, even relationships because it takes a lot more dedication.”

However, 8 of these 16 participants (50%) specifically indicated that the social downsides to their relatively heavy engagement in their sport were offset, and not as “negative” as they seemed, due to supportive friends and family. As Participant 22 (five to seven times per week) shared, “Of course, this commitment takes away from family time ... The lucky thing is, is that I met a [life] partner who thinks the same way that I do.” Participant 5 (five to seven times per week) added, “I have a good family. Everybody understood that I like to do this.”

## Discussion

The findings revealed no apparent variation in the psychological benefits experienced by older athletes who train and compete as little as one to two times per week, three to four times per week, and as much as five or more times per week. Overall, participants indicated a very positive psychological experience or benefits gained from masters sport, regardless of how often they were engaged on a weekly basis. Hours per week was significantly associated with frequency of engagement, and so it may be expected that perceived benefits hold for this factor as well. Masters athletes spoke about having less stress, greater self-confidence, and a sense of pride as a result of their participation. These positive aspects are an important part of successful aging (Geard et al., 2017), with evidence that well-being and self-achievement contribute to successful aging (Cho, 2002; Bowling, 2007; Geard et al., 2017, 2018). The psychological benefits of masters sport participation have been indicated in previous research (Ogles and Masters, 2000; Dionigi et al., 2011; Eime et al., 2013). However, the current study provides insight specifically into the older masters athlete (aged 50+ years) experience with evidence that suggests that these benefits may be realized regardless of time committed.

Similar to previous work (Lyons and Dionigi, 2007; Dionigi et al., 2011; Young, 2013), we also found that there were social benefits to participation in masters sport. Our study extends that work with evidence that these benefits were experienced similarly by older masters athletes across the different levels of frequency of participation. Whether participants were engaged from one to two times per week to five or more times per week, they experienced social benefits with both their families and friends. Athletes discussed a network of like-minded friends, which also extended beyond their sport with social gatherings among teammates outside of the sport environment, such as team meals or book clubs.

While social benefits were discussed by the masters athletes across all three groups of training and competition frequency and thus may be expected regardless of how often one is engaged, participants engaged five or more times per week were the only ones who discussed possible negative social consequences or downsides. They indicated missing family gatherings to compete or train and noted the toll it can take on one's family. They also described a shift in their friendship group, with their masters sport associates becoming a more important social circle. The loss or reduced time with friends outside their sport circle was seen as a downside of their engagement. This aligns with previous research which found that commitment to leisure time activity, such as running, was linked with perceived spousal conflict and constraints on families, especially those who are unable to adapt (Goff et al., 1997; Goodsell and Harris, 2011). Building on that work, our study suggests that there is a threshold of frequency of older masters athlete engagement, beyond which negative social consequences are likely to be experienced. Compromised family commitments and changed social circles may be expected with almost daily engagement or more. This may explain why some decrease or limit their masters sport participation or disengage completely.

However, while masters athletes who engaged in sport five or more times per week described the challenges to family commitments with greater time dedicated to their sport, several also indicated that this could be offset with supportive spouses and families. This aligns with the sport commitment model (SCM; Scanlan et al., 2003) which states that significant others can be either a social constraint on sport participation or a form of support. Adopting this model, Young and Medic (2011) found five sub-groups that influence the commitment of master's swimmers, with children and spouses likely to be both social supports and social constraints for older athletes' engagement. Some of the participants in our study indicated that their spouses were also masters athletes and that the shared training and similar experiences facilitated a greater understanding of their own commitment and compromised family time.

An apparent threshold of frequency of training and competition, beyond which negative social consequences may be experienced by older masters athletes, aligns with evidence of the direct association between frequency, time, and exertion and increased incidence of muscle fatigue, joint degeneration, and injury in this cohort (Maharam et al., 1999). Together, this work highlights that there can be too much of a good thing when it comes to masters sport. It is not surprising that there can be negative physical consequences of training and competition beyond a certain point (overtraining syndrome; Armstrong and Vanheest, 2002; Kreher, 2016), and our study adds to that understanding of masters athletes by revealing the negative social consequences that may occur beyond a certain point of engagement. Specifically, participants indicated that they needed to be able to miss (or give up) certain other aspects of life, such as time with friends and family outside of sport, to maintain their commitment to their sport. Importantly, however, our findings also suggest that the many positive psychological and social benefits of training and competition in masters sport may be realized with even minimal engagement.

There are several possible limitations of this study that must be noted, and which may provide directions for future research. Despite its many benefits, qualitative studies may be limited by self-reporting that is cross-sectional at a given point in time and influenced by social desirability bias (Brewer et al., 2011). Participants in this study may have been biased, for example, by particular benefits or negative consequences that they had very recently experienced at the time of the interview, or by a selective memory of the upsides of masters sport engagement. In addition, we relied on subjective measures of training and competition time, frequency, and intensity and participants may have overestimated or underestimated their engagement levels (Busse et al., 2009). Future research may build on the findings here and utilize objective measures of training and competition time, frequency, and intensity. This would allow for the determination of more nuanced patterns and thresholds of engagement for perceived benefits and downsides of masters sport participation.

Future research may also explore masters athletes' experiences specific to training and to competition (cf. Dionigi and O'Flynn, 2007), including any possible effect of differences in perceived exertion as identified here. Our study did not delve into those different contexts separately, yet nuances regarding psychological and social experiences in each may be expected. Variation by sport modality (e.g., aerobic, anaerobic, strength, and power; individual, paired, and team; competitive and recreational) should also be considered in future work, as type of sport may be a meaningful factor in frequency and intensity of training and competition and for masters athletes' experiences (Kusy and Zielinski, 2015). Additionally, the apparent impact of frequency of training and competition on social relationships outside the sport environment warrants further examination to better understand the role that, for example, family support plays in offsetting negative social consequences.

The findings of our study provide new insight into the older masters athlete's experience in sport with regard to time and frequency of training and competition, and perceived intensity of engagement. That common benefits can be experienced regardless of frequency of engagement is an important consideration for the design and promotion of sport for older adults. The findings also provide a springboard for further research to better understand features of masters sport that may ensure it is a positive part of successful aging through social connections, increased confidence, and resilience.

## Data Availability Statement

The raw data supporting the conclusions of this article will be made available by the authors, without undue reservation.

## Ethics Statement

The studies involving human participants were reviewed and approved by Western University Research Ethics Board. The patients/participants provided their written informed consent to participate in this study.

## Author Contributions

All authors have contributed equally to this work.

## Conflict of Interest

The authors declare that the research was conducted in the absence of any commercial or financial relationships that could be construed as a potential conflict of interest.
